# Process evaluation of prevention in long-term care facilities – resident and therapist perspectives on the benefits and acceptance of preventive speech and swallowing sessions

**DOI:** 10.1186/s12877-025-06822-8

**Published:** 2025-11-28

**Authors:** Wenke Walther, Isabell Fesser, Klaus Hager, Simone Miller

**Affiliations:** 1 Institute of General Practice and Palliative Care, Hannover Medical School, Carl- Neuberg-Straße 1, Hanover, 30625 Germany; 2 Experimental Phoniatrics of the ENT-Department, Hannover Medical School, Carl- Neuberg-Straße 1, Hanover, 30625 Germany

**Keywords:** Prevention, Swallowing, Language, Qualitative and quantitative research, Nursing homes

## Abstract

**Background:**

Current demographic trends make the prevention and delay of diseases and their consequences particularly relevant for older adults. A German intervention study developed a programme to prevent swallowing difficulties and language decline among individuals residing in long-term care facilities. As part of the process evaluation, this study assessed residents’ satisfaction with this programme and the extent to which therapists considered the exercises feasible and beneficial for the target group.

**Methods:**

A mixed-method process evaluation was conducted. Participating residents completed a Likert-scale questionnaire to assess their satisfaction with the intervention, its organisational aspects and materials, volitional devices, and their personal success. Quantitative data were analysed descriptively using SPSS. Furthermore, seven qualitative interviews were conducted with the speech-language therapists (SLTs) who administered the sessions. These interviews were analysed using Mayring’s method of content analysis, with MAXQDA software. Subsequently, a comparative analysis was conducted between the qualitative and quantitative findings.

**Results:**

All 56 participating residents (86% female, mean age = Ø 82 years) reported high levels of satisfaction. The SLTs demonstrated a high degree of adherence to the intervention manual and reported positive impacts on participants’ performance in swallowing and semantic exercises, as well as on the transfer of these exercises into daily life. Additionally, participants were described as having increased awareness of their limitations in swallowing and speech. Potential improvements were identified with regard to the intervention materials, group composition, and organisational aspects.

**Conclusion:**

Residents expressed satisfaction with the preventive intervention, which SLTs described as both feasible and beneficial. However, adjustments would need to be made to accommodate the target group’s general impairments. Furthermore, positive group effects were observed, including enhanced communication, reduced loneliness and greater social participation.

**Trial registration:**

The trial was registered with DRKS (German register for clinical trials) in 27,062,023 (study ID DRKS00031594) and the WHO International Clinical Trials Registry Platform (secondary register).

**Supplementary Information:**

The online version contains supplementary material available at 10.1186/s12877-025-06822-8.

## Background

Current demographic trends are rendering prevention among older adults increasingly relevant. The proportion of individuals over the age of 65 is steadily rising, having reached 22% in 2022, and is projected to grow to 29.7% in Germany by 2065 [1]. Preventive services aim to avoid or delay illnesses and their consequences, including the onset of care dependency and limitations to health. A key goal of age-related prevention is the maintenance of independence and social participation [2]. The World Health Organization (WHO) defines ‘healthy ageing’ as the process of developing and maintaining the functional abilities necessary for well-being in later life [3]. This goal may be challenged by age-related muscular and cognitive decline, which can affect swallowing, speech-language and broader cognitive functions.

Reduced swallowing functions can result in malnutrition and dehydration, thereby diminishing quality of life [4]. Ageing is often accompanied by a decline in muscle strength, which affects the muscles of the mouth, throat and larynx as well as the necessary protective reflexes [5]. Dysphagia is diagnosed in 50–60% of all residents in long-term care facilities, and in people over 85 years, pneumonia is the direct cause of death in 15–20% of cases [6]. These figures underscore the significant need for preventive measures that benefit both older people and the healthcare system in general in the form of potential cost savings.

Moreover, communication is essential for meeting personal needs, as well as for maintaining psychological well-being and sustaining cognitive abilities [7]. Contrary to the assumption that daily language use leads to well-developed language skills over the course of a lifetime, words are not so easily remembered in old age and it takes longer to process language [8]. However, opportunities for communication often decline with age, and feelings of loneliness tend to increase [9]. Among older adults residing in long-term care (LTC) facilities, daily experiences and communication are frequently limited to routine activities. Diminished cognitive processes, such as memory management and attention, may also affect language skills [10]. For this reason, language difficulties in old age may serve as an early indicator of dementia [11].

The ‘Guidelines on prevention in LTC facilities’ [12] outline a range of preventive goals, including those related to cognition, mental well-being and nutrition. Typically, such measures encompass care provided by the facility, supportive activities and strategies that residents can adopt independently to preserve their functional abilities. However, only few interventions aim specifically at the maintenance of swallowing function and the prevention of communication loss or semantic-lexical decline. In accordance with the WHO’s objective of promoting ‘healthy ageing’, this discrepancy was addressed through the development and implementation of a preventive intervention programme for *orofaciopharyngeal and linguistic-communicative activation in older age* (OrkA).

The aim of this programme was to support safe food intake by maintaining swallowing ability, while also enhancing semantic-lexical functioning and promoting communicative participation [12]. A cluster randomised controlled trial (cRCT) was conducted in order to investigate these objectives in older adults. To establish and improve this preventive intervention, the present study assessed residents’ satisfaction with the programme as part of the process evaluation as the primary outcome. Additionally, the evaluation assessed the speech and language therapists’ (SLTs) complementary perspectives on the programme’s perceived feasibility and usefulness. Both approaches are necessary to develop and enhance this preventive intervention.

## Methods

The process evaluation in the OrkA intervention study was conducted using a mixed-method design. The process evaluation is based on the consolidated framework for implementation research (CFIR) [13]. Three of the five main domains were taken into account in this manuscript: the outer setting in the form of resident satisfaction to identify needs and barriers, the inner setting in the form of interviews with the SLTs to identify organisational structure and communications within the LTC 

facilities, and characteristics of the OrkA intervention in both forms of the survey. Characteristics of the individuals and the process of implementation has not yet been taken into account, as the implementation phase has not yet taken place.

Recruitment took place in two stages. First, LTC facilities were recruited via supporting organisations, and in a second stage, residents were approached by care staff to participate. A comprehensive examination was carried out before the residents were included into the study (Table 1). Detailed information regarding the recruitment strategy, as well as the inclusion and exclusion criteria, can be found in the study protocol [12]. Prior to participation, all respondents were informed about the study objectives and the voluntary nature of their involvement, and written informed consent was obtained. The research was approved by the ethics committee of Hannover Medical School (No. 10650_BO_S_2022) and conducted in accordance with the Declaration of Helsinki.

**Table 1:** The inclusion and exclusion criteria of the participants

The inclusion criteria	The exclusion criteria
• aged 60 years or older • residing in a LTC facility • no mild or moderate frailty (i.e., Clinical Frailty Scale: level < 5) [14] • able to give informed consent • sufficiently able to hear (i.e., 80% speech comprehension at an everyday speech volume)	• a current diagnosis of a swallowing, speech, and/or language disorder and an associated prescription • suspected aspiration (i.e., EAT-10 score > 2) [15] • moderate or severe dementia (i.e., DemTect ≤ 8) [16] • estimated life expectancy of < 6 months, based on the “Double Surprise Question” [17]

The intervention comprised 24 training sessions delivered over 12 weeks, with two 60-minute group sessions held weekly within LTC facilities. Each session followed a consistent structure:

1. a short mobilisation segment designed to activate the entire body and promote an optimal training posture.
2. two orofaciopharyngeal exercises aimed at preserving swallowing ability (e.g., exercises for elevating the larynx, strengthening the lips and improving tongue mobility). To achieve these goals, volitional devices such as spatulas, buttons, beads, suction tubes, etc. were used. According to the Rehabilitation Treatment Specification System, all of the used devices are classified as volitional, as they required active use and involvement by the residents. Non-volitional devices, which are passive and do not require active engagement, were not used [18].
3. two language exercises targeted the semantic-lexical domain (e.g., exercises for categorisation, sematic feature analysis and synthesis of word forms).
4. the language exercises were further augmented through free-flowing biographical conversation.
5. concluded with the group (i.e., participants and the trainer) singing well-known songs together – a practice intended to support the memorisation of lyrics and melodies while activating the laryngeal source.

Separate from these sessions, the programme also included a self-directed learning module, featuring exercises from the sessions that residents could complete independently. These exercises – referred to as voluntary homework (VHW) – were compiled as worksheets in a separate folder, and they included free-form conversations related to the prior session’s topic.

For the quantitative component of the process evaluation, participating residents were asked to complete a questionnaire at the end of the 12-week intervention to assess their satisfaction with various organisational, content-related, expectation-related and outcome-related aspects, using a 6-point Likert scale ranging from 0 (*not at all satisfied*) to 5 (*very satisfied*) [19]. Residents were also invited to suggest potential improvements as free-text comments. Furthermore, residents’ frequency of participation and engagement with the VHW was documented. These data – including participation rates, VHW completion and questionnaire responses – were evaluated using descriptive statistics based on frequencies using SPSS (version 29.0). In addition, total scores were calculated for variables grouped by topic (organization, swallowing, speech). To analyse group comparisons between these topics, non-parametric tests (Wilcoxon rank sum test for paired samples) were used for ordinal data. Spearman’s rank correlation coefficient rho (r_s_) was used to determine correlations between ordinal data and Pearson´s correlation coefficient (r) for metric data. The statistical significance level was set to 5%. The free-text responses were analysed using content analysis [20].

For the qualitative component of the process evaluation, semi-structured interviews were conducted with the four SLTs who implemented the intervention, following the conclusion of each intervention phase. As the SLTs were already part of the study team, no separate recruitment was necessary. Invitations were extended via email and in person, and all SLTs were informed about the voluntary nature of their participation and the study objectives. The aim of this approach was to gather comprehensive insights into the experiences of each intervention group [21].

The semi-structured interview guide was developed in accordance with the content of the intervention and the broader research interests. The evaluation of the programme requires data from self-reports rather than quantitative measurement parameters. The aim was to examine the experiences, challenges and decisions of therapists in a field that is still unknown (prevention in speech therapy for older people). In semi-structured interviews, a guideline provides direction while still offering space to talk about experiences. The guide for the interviews with speech therapists was designed to enable a quick start, covering both general and more detailed questions. The content was based on the CFIR [13] and ranged from communication with the institutions to the feasibility and impact of the programme and SLTs skills. Five thematic categories were defined: (1) general experience with the intervention; (2) intervention progress, including questions about the organisation and specific content; (3) suitability of the material; (4) perceived benefits (i.e., observed effects); and (5) final questions regarding possible adjustments (see supplement 1). Interview questions were formulated as narrative prompts, with optional follow-up and expansion questions to encourage open responses. This interview guide was pilot tested and subsequently used in interviews by a maximum of two experienced female researchers (SLTs) with doctoral qualifications (WW and SM). Interviews were held between December 2023 and March 2025 in quiet meeting rooms at the research institute and were recorded as audio files. One SLT managed multiple groups concurrently and therefore reported on two groups during each interview. Additionally, a tandem interview was conducted with two trainers simultaneously. With the exception of a single notetaker during the tandem interview, no other individuals were present. Interviewers also took field notes. All interviews were transcribed verbatim with minimal language and punctuation editing for clarity, and SLT anonymity was preserved throughout (T1 = trainer 1, T2 = trainer 2, T3 = trainer 3, T4 = trainer 4). Transcripts and findings were not returned to participants for review or correction.

The qualitative evaluation was conducted using MAXQDA software [20], following the content analysis method developed by Mayring [22]. Initially, IF defined super- and subcategories categories deductive from the interview guide, which were then inductive refined through a review of the material. These categories were then summarised into a code tree. Subsequently, IF and WW conducted a trial coding of approximately 20% of the data, yielding an intercoder agreement of 67.9%. Discrepancies were discussed and recoded, with the code tree revised accordingly. A second round of coding resulted in an improved intercoder reliability of 97.1%. Finally, IF applied the final coding scheme to the entire data set using MAXQDA (24.7).

## Results

### Primary results

#### Facility and resident characteristics

The quantitative analysis was based on 56 completed questionnaires from residents across 10 LTC facilities. The number of participants per facility ranged from three to nine. Facilities had an average capacity of 104 care places (range: 36–201). Of these, five were operated by private providers, one by a public institution and four by non-profit organisations. On average, the facilities offered 6.6 (± 2.3) activities for residents. A median of 7.5 staff members (range: 0–15) from the accompanying services were involved in delivering these activities, which included: sports, reading and cognitive training (in nine facilities); singing (in eight facilities); gardening, handicrafts and cooking (in seven facilities); and dancing (in three facilities). Occasional activities included concerts, bingo, film screenings and bowling.

Participating residents were predominantly female (85.7%), with an average age of 82 years (± 8.7, min.-max.: 61–99 years). The majority were either single or widowed (80.3%), and 75% reported regular contact with close relatives. Regarding educational background, 57.1% had obtained a middle school leaving certificate and 41.1% had completed vocational training. The median care level, as defined by the German compulsory nursing care insurance scheme, was 2.5 (range: 0–5). On average, residents had 2.5 (± 1.6) medical diagnoses. 36% of these were related to swallowing and/or speech disorders. The mean number of daily medications was 6.6 (± 3.5), and 54% were taking medications unrelated to speech-language and/or swallowing functions. Table 2 presents a comprehensive overview of the characteristics of participating residents.

**Table 2:** Characteristics of participating residents

Resident characteristics	*n* = 56	
Gender: female	48	(85.7%)
Age: mean (*SD*)	82	(8.7)
Life situation		
single	12	(21.4%)
widowed	33	(58.9%)
married/partnership	9	(16.7%)
missing information	2	(3.6%)
Level of school education		
middle school	32	(57.1%)
high school	9	(16.1%)
missing information	15	(26.8%)
Level of qualification		
no completed training	13	(23.2%)
vocational training	23	(41.1%)
college/university	4	(7.1%)
missing information	16	(28.6%)
Regular contact with relatives: yes	42	(75.0%)
Care level (according to the German compulsory nursing care insurance scheme)		
no care level	11	(19.6%)
grade 1	2	(3.6%)
grade 2	15	(26.8%)
grade 3	21	(37.5%)
grade 4	6	(10.7%)
grade 5	1	(1.8%)
Number of medical diagnoses: mean (*SD*)	2.5	(1.6)
heart disease	32	(57.1%)
kidney disease	11	(19.6%)
cancer	9	(16.1%)
dementia	7	(12.5%)
status post stroke	6	(10.7%)
Number of multimorbid residents (≥ 3 diseases):	23	(41.1%)
Number of medications: mean (*SD*)	6.6	(3.5)
Number of residents with polypharmacy (≥ 5 medications)	43	(76.8%)

The mean participation rate in the intervention sessions was 20 out of a possible 24 sessions (± 4.9) with a minimum of 3 participations. Furthermore, residents engaged in the VHW exercises designed to maintain swallowing function and speech-language ability an average of 109 (± 161.4) and 15 times (± 7.4), respectively, between sessions. On average, three (± 3.4) informal conversations were held concerning group-related topics as part of the VHW. Participation in intervention sessions exhibited a strong positive correlation (*r*: 0.697; *p*: <0.001) with engagement in additional speech-language VHW.

#### Resident satisfaction

**Fig. 1:**
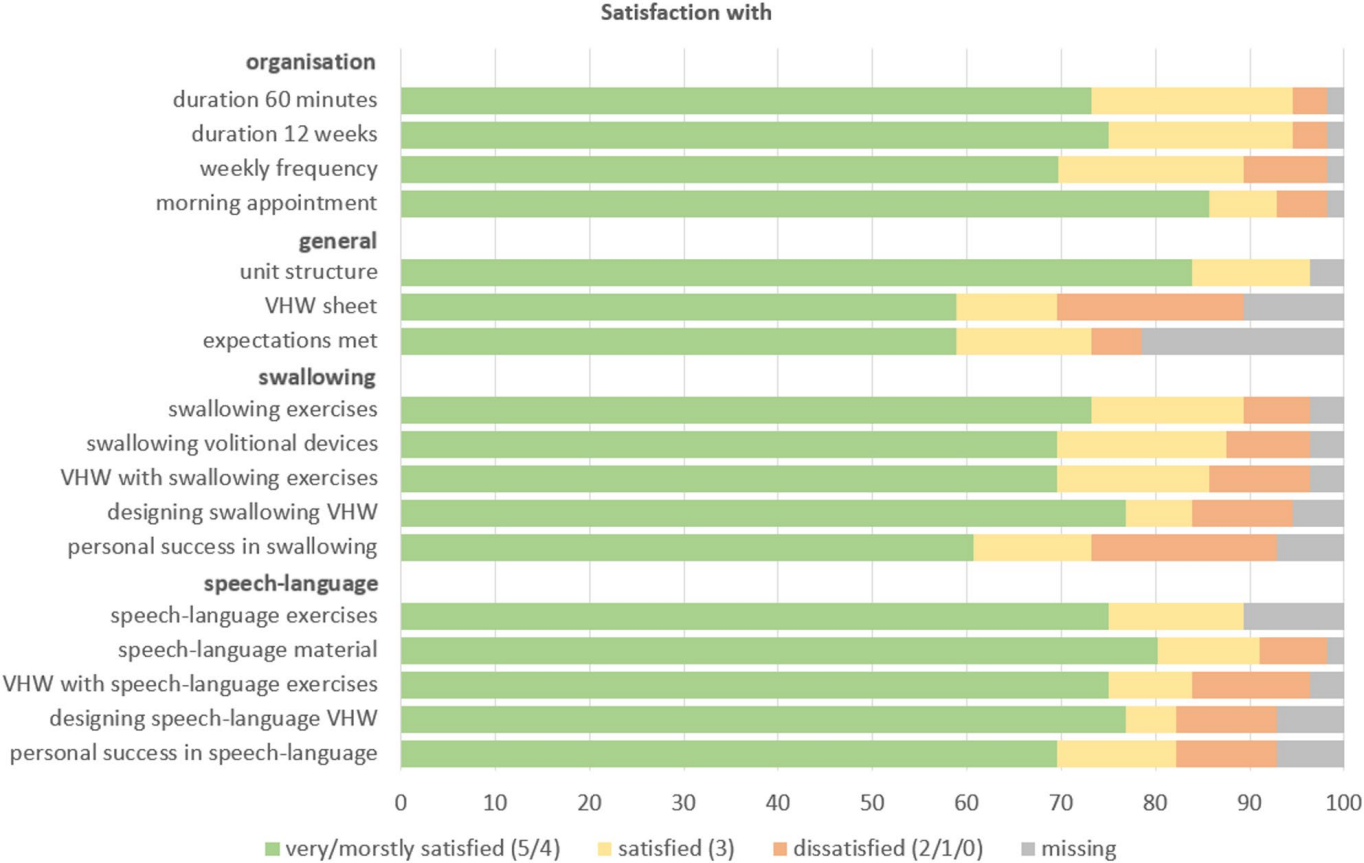
Residents’ satisfaction with the intervention in percent *n* = 56 (VHW = volunteer homework)

Residents’ overall satisfaction with the intervention was high (see Fig. 1). Satisfaction with the organisational aspects of the programme received in all four aspects a median score of 4 (min.-max.: 1–5) on the 6-point Likert scale (0 = not at all satisfied; 5 = very satisfied). However, 60.7% of residents expressed a desire to modify the standard 60-minute session length. Specifically, seven participants indicated a preference for shorter sessions, recommending 30 min (*n* = 1) or 45 min (*n* = 5). In one instance, a resident merely described sessions as ‘a bit too long for me’. Conversely, four participants expressed a preference for longer sessions, with one participant specifying 90 min as a more appropriate length and three asking for sessions that were ‘a bit/partly rather longer’. The remaining 20 residents expressed satisfaction with the 60-minute sessions.

Regarding the overall intervention, six residents expressed a preference for a shorter duration (6–10 weeks), while another six indicated a preference for a longer duration (3–6 months). Furthermore, 15 residents considered the intervention duration appropriate, claiming that ‘the duration of the training was just right’. Concerning the frequency of sessions, 16 residents found the twice-weekly schedule suitable (‘is good’), while one expressed a desire for more frequent sessions (‘3+’) and 12 indicated a preference for only one session per week. 22 residents deemed the scheduled training time (typically 10:00 a.m.) appropriate. Only four expressed a preference for an earlier start, while three indicated a preference for afternoon sessions.

Overall, 84% of participants reported a high level of satisfaction (very or mostly satisfied) with the session structure, yielding a median score of 4 (range: 3–5). A total of 59% reported satisfaction with the VHW component, while 20% expressed dissatisfaction. Similarly, 59% indicated that the intervention met their expectations (very or mostly satisfied). Notably, 21% did not respond to this question, possibly due to a lack of clearly defined expectations. In the free-text responses, 35 residents provided similar explanations. Their expectations were generally modest, often limited to hopes for pleasant interactions and opportunities to form new social connections. However, the unfamiliar nature of the intervention activities contributed to a degree of uncertainty. Participants expressed a desire to familiarise themselves with the exercises, and described the experience as marked by constructive collaboration. (‘I had no expectations beforehand apart from a nice group and getting to know people’; ‘none, as it was all new territory’; ‘I just wanted to get to know exercises’; ‘good cooperation’).

In summary, residents reported slightly but statistically significant lower satisfaction with the swallowing domain (content, volitional devices, VHW, exercise sheets, perceived success) (total score Ø 19.7 ± 3.81) relative to the speech-language domain (total score Ø 20.3 ± 4.46; *p* = .033). The survey results revealed that 60% of residents expressed high satisfaction with their personal success in swallowing-related activities. However, free-text responses often noted no prior difficulties with swallowing.

Overall satisfaction was strongly correlated with satisfaction regarding the organisational aspects (*r*_*s*_: 0.812; *p*: <0.001), the intervention materials (*r*_*s*_: 0.900; *p*: <0.001), the VHW (*r*_*s*_: 0.822; *p*: <0.001) and perceived success (*r*_*s*_: 0.909; *p*: <0.001). A modest positive correlation was identified between satisfaction with the organisational aspects and the number of care places in the LTC facility (*r*_*s*_: 0.277; *p*: 0.042). Additionally, resident age showed a positive correlation with satisfaction with the materials (*r*_*s*_: 0.335; *p*: 0.019). Conversely, satisfaction with one’s personal success was negatively correlated with the number of medical diagnoses (*r*_*s*_: − 0.333; *p*: 0.029).

### Complementary perspectives of the speech and language therapists

A total of seven interviews were conducted, encompassing 11 instances of intervention delivery. The average interview duration was 55 min (range: 39–112 min). The participating SLTs, all of whom were involved in the OrkA project, were aged 28 to 39 years at the time of the interviews. Three held a master’s degree and one a bachelor’s degree. All had practical experience in the treatment of individuals with speech, language, swallowing and voice disorders. One had prior experience delivering preventive group programmes in the field of voice training, while two had experience with curative group therapy (one in neurorehabilitation and the other in developmental speech-language disorders).

To address the research question, two main categories (MC) from the established coding system were considered: (1) manual fidelity and challenges and (2) impact of the intervention.

#### Main category: manual fidelity and challenges

The programme’s feasibility was evaluated by examining adherence to the self-learning module and any challenges (-) alongside its fidelity to the manual (+). Due to the saturation of the content the MC was further divided into subcategories (SC).

**Fig. 2:**
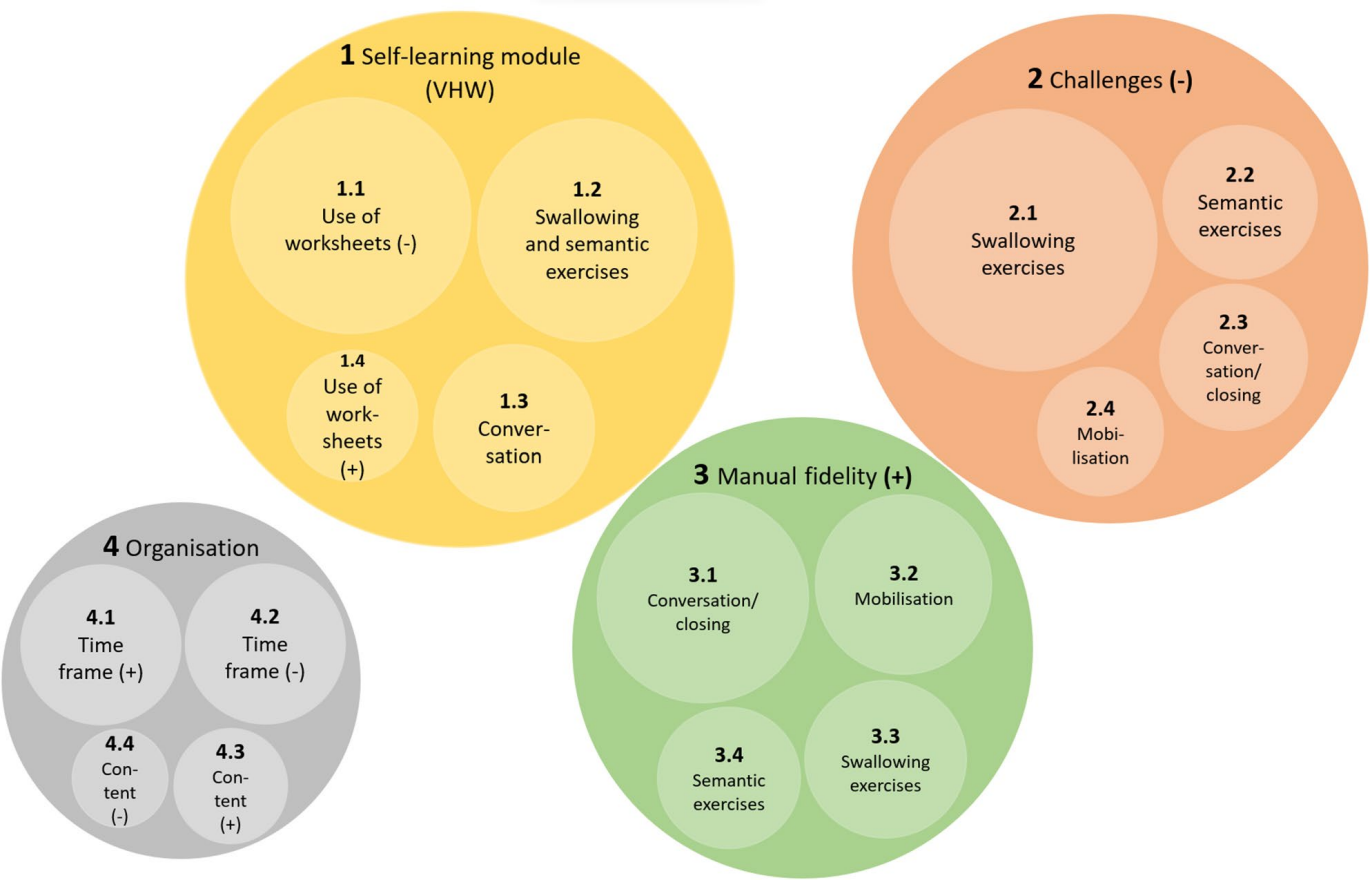
Illustration of the coding frequency of the upper and lower categories for manual fidelity

Figure 2 provides a visual representation of the frequency with which the various categories were coded during the interviews. The diameter of each circle is proportional to the frequency of the code assignment. The results are organised by frequency of coding.

#### Self-learning module

SLTs provided feedback on the VHW and materials (worksheets), describing that small font sizes, difficult-to-see illustrations or unclear wording posed challenges for residents. In certain cases, residents misused the worksheets, simply forgot or misplaced their folders (SC 1.1). However, for others, the folder proved an effective organisational system and a valuable resource for independent practice (SC 1.4).

*Well*,* I liked the folders and also the residents liked them. And although a few things were forgotten here and there*,* it wasn’t a big deal. (I2*,* T3)*

SLTs’ feedback on the appropriateness of VHW (SC 1.2) focused on exercises that could be integrated into everyday routines and completed without extra volitional devices.

*Yes*,* exactly*,* the residents enjoyed doing exercises that they could easily do in between other activities*,* for example while going for a walk or watching TV. (I1*,* T2)*

All SLTs agreed that the semantic-lexical self-learning exercises were positively received. This was largely attributed to the residents’ well-known fondness for familiar formats such as riddles and word games.

*I’ll jump right in there; the letter salad and the search puzzles were particularly popular with everyone when it came to homework. (I1*,* T2)*

In contrast, the conversation task was seldom implemented, primarily due to the fact that the task was not included on the worksheets but expressed only verbally by the SLTs (SC 1.3).

#### Challenges (-)

The interview data revealed numerous challenges pertaining to the implementation of the exercises, particularly those aimed at maintaining swallowing function (SC 2.1). For instance, while some residents preferred exercises involving volitional devices, such devices often posed problems due to age-related physical limitations. In certain instances, residents chose to avoid using volitional devices altogether.

*I’ve just mentioned osteoarthritis […] declining fine motor skills*,* which also makes it difficult to use assistive devices. (I4*,* T1)*

In addition, the widespread use of dentures among participants hindered the execution of intraoral exercises (e.g., involving the tongue, palate or velum), making certain tasks particularly challenging.

*What made it more difficult was that some of the residents had dentures*,* which made the suction exercises more difficult*,* for example. (I2*,* T3)*

A specific challenge noted by all SLTs was the use of laminated objects in exercises, as these were difficult to suck up from a smooth surface using a straw.

*Exactly*,* and then it’s so smooth […] because of the surface texture*,* and I know from experience that sometimes*,* if the suction isn’t strong enough*,* it sticks a little bit. (I2*,* T3)*

Comparable challenges were reported regarding the language and mobilisation exercises (SC 2.2, 2.4). SLTs observed that the language exercises could be either too easy or too demanding, depending on the groups’ physical and cognitive conditions. In cognitively fit groups, for example, the exercises had to be adapted by increasing their complexity. In other groups, however, introductory explanations were needed before tasks could be mastered.

*Well*,* that was certainly one of the easier exercises*,* but when it came to rhymes*,* […] I first had to explain to the first group what a rhyme is. (I1*,* T1)*

Visual impairments also constrained participation, as some residents could not clearly perceive the language exercise materials or demonstrations.

*For the people who have […] visual impairments*,* it was also very difficult with some of the material*,* um*,* because they couldn’t read the text or couldn’t recognise the pictures. (I6*,* T4)*

Mobilisation exercises were generally reported as the least challenging (SC 2.4). Nonetheless, SLTs sometimes needed to adapt these exercises to suit the specific physical needs of each group.

*So*,* because there were two people who used a walker and one patient had a stump*,* it was sometimes only possible do these mobilisation exercises to a limited extent. (I2*,* T3)*

#### Manual fidelity (+)

According to the SLTs, the conversation round and session closing were most consistently implemented in line with the intervention manual (SC 3.1). In contrast, the swallowing exercises showed somewhat less adherence (SC 3.3). Nevertheless, several positive statements were expressed about these exercises, particularly those involving gustatory stimuli, which were reported to enhance residents’ enjoyment and provide a sense of recreation.

*So*,* these exercises with sucking in jelly string candy*,* were really great*,* they enjoyed them and eating jelly*,* too. (I2*,* T3)*

The language exercises (SC 3.4) comprised a balanced array of challenging and practical exercises. SLTs observed that residents generally responded positively to them.

*Yes*,* exactly*,* the rest was actually good*,* and I also found the material mostly appropriate in the sense that there are picture cards or colour cards that can be used. (I2*,* T3)*

Feedback on the initial mobilisation exercises was also largely positive (SC 3.2). SLTs noted various beneficial aspects of these exercises, including residents’ motivation, physical activation and improved posture, which prepared them for the subsequent swallowing exercises.

*However*,* I think that*,* for me*,* they are an indispensable part of the method if you want to do the orofacial exercises afterwards […] they always served as an icebreaker. (I1*,* T1)*

The extent to which specific conversation topics of the communicative activities (SC 3.2) were accepted depended on residents’ life experiences. Conversations concerning personal histories were conducive to the generation of ideas, though they occasionally evoked strong emotional responses.

*It can also be a very difficult and sensitive topic*,* the relationship with relatives*,* and when it suddenly comes out and you hear the reasons behind it*,* it can sometimes be deeply saddening to learn about these things. (I7*,* T1)*

Each session concluded with a group song, or poems if the song was rejected by the residents. According to SLTs, this activity was generally accepted and appreciated across most groups.

*But overall*,* we always did it*,* and I think it was always perceived as a nice way to finish*,* and they always liked about the texts. (I1*,* T2)*

#### Organisation

The overall duration of training sessions was confirmed by all SLTs as appropriate, despite group related variations (i.e., more explanation time).

*Well*,* I thought that was good because they had already had breakfast. Of course*,* there were always a few people who had a doctor’s appointment*,* but otherwise they were pretty much always there. So*,* I think that ensured that they were able to pay full attention. (I2*,* T3)*

Based on this feedback, the training frequency should be reduced to one session per week, in order to accommodate both weaker residents and those with demanding schedules. While session structure and content were less frequently discussed in the interviews, SLTs generally viewed them positively (SC 4.3, 4.4).

#### Main category: impact of the intervention

The second MC addressed the perceived impact of the intervention from SLTs’ perspective.

#### Learning and behavioural impact

**Fig. 3:**
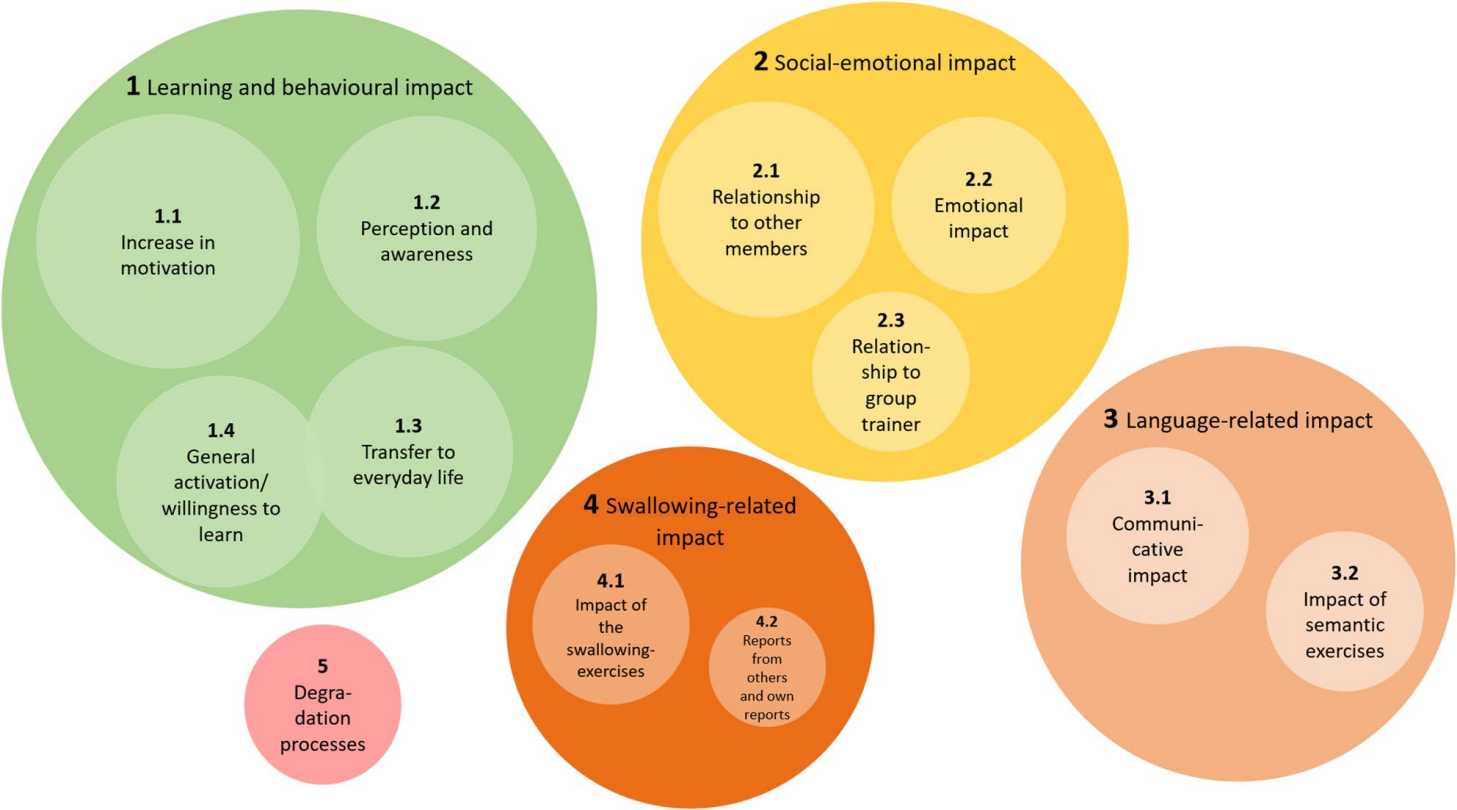
Illustration of the coding frequency of the super- and subcategories related to the observed impact of the intervention

This category received the highest number of codes (see Fig. 3). SLTs frequently observed an increase in residents’ motivation (SC 1.1), characterised by greater participation, increased self-study and heightened involvement in group sessions.

*Hm*,* well*,* I would say yes. The residents who stayed until the end definitely did*,* because they had that as motivation to stay until the end*,* or not only that*,* but also because they wanted it to continue. (I1*,* T2)*

Residents’ awareness (SC 1.2) of their own and others’ speech and/or swallowing difficulties appeared to increase. They also developed a better understanding of extra- and intraoral muscular structures and physical states of tension, and came to recognise their functional significance.

*Or sometimes the residents really realised*,* ‘Oh*,* I really can’t do that right now’*,* and then noticed their own little*,* smaller or larger deficits. So*,* yes*,* so it was definitely very valuable overall*,* yes (I1*,* T2).*

SLTs reported that some residents expressed a desire to continue practising independently after the 12-week programme. Several incorporated specific exercises into their morning routines to maintain swallowing ability. Others utilised the language-related worksheets to foster social interaction (SC 1.3 transfer in everyday life).

*And then there were others who really put together a morning exercise programme with their favourite exercises*,* which they integrated into it. (I4*,* T1)*

Even residents who expressed initial scepticism demonstrated a degree of receptiveness to the intervention, as evidenced by their willingness to engage in and complete the training.

*But apart from that*,* I think they handled it well because they also knew: ‘Okay*,* this is just the programme*,* this is what’s been decided*,* and I’ll do it in some way or other’. (I5*,* T3).*

#### Socio-emotional impact

Over time, the residents became more open with one another (SC 2.1), and developed greater trust in the SLTs (SC 2.3). In certain instances, residents met with one another outside of scheduled sessions.

*And I think*,* from what I’ve noticed*,* that they didn’t know each other that well before and so of course they got to know each other better. They also talk in private now. (I2*,* T3)*

SLTs also observed emotional changes during the intervention (SC 2.2). These included signs of improved mood, enhanced self-confidence and greater willingness to demonstrate one’s abilities to the group.

*It’s really nice when they somehow become the centre of attention for a moment because they somehow can sing a song by heart or recite a poem by heart. (I1*,* T1)*

#### Language-related impact

SLTs found it challenging to assess the extent to which the language exercises (SC 3.1) resulted in significant content-related improvements. However, some residents were observed to apply word-finding strategies over time.

*In any case*,* during one session I definitely noticed that one resident was consciously using word-finding strategies*,* which was not the case before. (I1*,* T2)*

Additionally, SLTs noted a perceived improvement in communicative engagement (SC 3.2), as evidenced by residents’ increased ability and willingness to exchange ideas during group discussions.

*Yes*,* and I also found it really nice to see that some of the participants gained more confidence in engaging in conversation during those twelve weeks. (I6*,* T4)*

#### Swallowing-related impact

With regard to the swallowing exercises (SC 4.1), SLTs observed marginal improvements in residents’ movement coordination and range of motion during repeated practice.

*But when comparing the myofunctional exercises*,* for example*,* when we did the jelly string suction exercise for the first time and then again later*,* I noticed that it was easier for them*,* that there was a change in terms of the range of motion. (I2*,* T3)*

Moreover, SLTs reported that some residents noticed personal improvements. (SC 4.2) For instance, one resident reported fewer incidents of choking while drinking water at night.

*However*,* she generally believes that the exercises have also helped*,* and yes*,* she has less difficulty drinking*,* especially at night. (I1*,* T2)*

#### Degradation processes

In some cases, SLTs observed deterioration in residents, despite their participation in the intervention. Degradation processes included cognitive decline, depressive symptoms and a worsening of physical limitations, resulting in increased reliance on nursing care or assistive devices.

*Sometimes the wheelchair was necessary because the lady suffered a few health setbacks during the intervention period*,* or simply deteriorated*,* also mentally. (I7*,* T1)*

## Discussion

Residents expressed a high level of satisfaction with the intervention, and in many cases, SLTs were able to implement the sessions in accordance with the manual. The intervention appeared to have a positive impact on not only residents’ swallowing and speech-language abilities, but also their willingness to engage with others and communicate within the group setting. Nonetheless, several areas within the intervention and VHW material, as well as certain organisational aspects, were identified as having the potential for adaptation and improvement.

Notwithstanding the high satisfaction expressed by residents, the exercises designed to maintain swallowing abilities in general emerged as the most critical component in the process evaluation of the training programme. Feedback from both residents and SLTs clearly indicated that some of the swallowing exercises, volitional devices (e.g., spatula, suction objects), and design of the self-learning module would benefit from further revision and more precise tailoring to the needs of the target group. The evaluated difficulties of the muscular swallowing exercises could also be due to the fact that not all parts of pre-practice were fulfilled, which is one of the principles of motor learning. The aims of pre-practice include motivating people to learn the task, providing them with the correct information on how to perform the task, and modelling the task. In order to ensure motivated performance, people must understand its importance [23]. The critical attitude towards swallowing exercises may be due to a lack of understanding of their importance for prevention. This is also reflected in residents’ low expectations of the programme.

A previous study of Hägglund [24], which examines effects of oral neuromuscular training on swallowing dysfunction has indicated that dropout rates and reasons for withdrawal often reflect the difficulty participants experience when engaging in oral exercises involving volitional devices [24]. At the same time, other studies have demonstrated that resistance-based exercises utilising volitional devices such as spatulas can result in measurable improvements in tongue strength, which in turn may enhance bolus control and swallowing function [25]. Furthermore, Namiki et al. [26] found that tongue-pressing exercises against resistance may help to prevent dysphagia associated with aged-related sarcopenia.

In the present study, the use of volitional devices, was found to increase motivation and enjoyment among certain participants. This suggests that offering exercise with and without volitional devices may help to accommodate individual preferences and physical capacities. Notably, suction exercises using a straw or requiring tongue-palate suction presented considerable difficulty, due both to residents’ physical limitations and the properties of the volitional devices used. For example, laminated suction devices were often too smooth or asymmetrical, making them difficult to manipulate with a straw. Additionally, inadequate suction force or poor posture often hindered execution. Dentures also posed a barrier to intraoral exercises, particularly those involving tongue-palate suction. In this regard, the provision of alternative suction exercises targeting the same muscle groups may prove beneficial. For instance, tongue press exercises, which are easier to perform with dentures, have been shown to effectively promote anterior tongue elevation. In addition, Namiki et al. [26] reported that a four-week protocol of tongue-pressing (10s press, 10s rest, five repetitions, twice daily) significantly improved hyoid elevation while reducing aspiration, penetration and pharyngeal residue in cases of presbyphagia. Suction exercises aimed at training velum elevation should also be adapted, particularly with regard to the volitional devices used (e.g., symmetrical suction devices).

SLTs reported that language exercises had a predominantly positive effect on residents’ abilities. However, a primary challenge related to the material design. In some cases, the font was either too small or lacked sufficient contrast, making it difficult for residents to read. Furthermore, the illustrations were often identified as blurred, too small or otherwise difficult for residents with visual impairments to recognise. Residents’ attempts to cope with these barriers may have increased their cognitive load, which recent research suggests should be minimised in older adults [27]. Consequently, the language materials should be re-designed to reduce distractions and ensure optimal clarity and accessibility. In particular, older adults have been shown to prefer larger fonts, strong contrast, high-resolution images and age-appropriate instructions [27]. Hill-Briggs and Smith [28], in their evaluation of educational materials for diabetes and cardiovascular disease, found that criteria such as sentence length, font size, word choice, visual design and overall comprehensibility were frequently unmet. Further research is required in this area to inform the development of language training materials that effectively support linguistic and cognitive function in older individuals experiencing age-related visual impairments.

With regard to group dynamics and associated processes, the initial group meeting should incorporate a relaxed introduction, accompanied by a clear and comprehensible explanation of programme objectives that is appropriate for the specific group [29]. Support from the group leader may be especially important during the initial phase of group formation (exploration and orientation), at which point participants typically experience heightened insecurity [29]. Thus, it is recommended that the group leader provide opportunities for participants to articulate their individual goals and expectations in small group settings, while also allowing time for social interaction [29]. In terms of expectations, it would be beneficial to present a clearly defined set of intervention goals at the outset, to facilitate a collaborative and goal-oriented training environment. The establishment of a shared objective may also contribute to the development of group cohesion and solidarity [30].

While SLTs would have liked to have extended the sessions, this viewpoint was not shared by residents. One potential explanation for this divergence lies in the heterogeneity of the groups. Some were significantly larger (i.e., with eight residents participating), which necessitated a greater allocation of time. In addition, some groups included residents with more complex visual and/or mobility impairments, who required further support. Another potential explanation is that the scope of each session was too ambitious for the 1 h allotted, and SLTs were intent on adhering to the manual. A simple adaptation could be to administer a single semantic-lexical exercise, while reserving a second exercise for more advanced groups. However, extending the duration of the intervention to 90 min, for example, may be impractical in an LTC facility, where the majority of sports and cognitive training programmes last between 45 and 60 min [31]. Furthermore, such an extension would not align with the preferences expressed by most residents. Rather, in accordance with the expressed preferences of 21.4% of the residents, the frequency of sessions might be reduced to once per week – an option that SLTs indicated would be feasible.

Nevertheless, the question remains as to whether this reduced frequency would be sufficient. Studies have demonstrated that, to achieve the desired level of adaptation, physical activity must exceed standard levels [32]. This requires a certain intensity – whether through mechanical or resistance load, repetition within exercises or sustained duration over time. It is therefore uncertain whether less frequent training would produce comparable outcomes and resident satisfaction, or whether the intensity of individual exercises would need to be increased. According to the residents, the 12-week programme could be extended to 3 to 6 months, or shortened to 6 to 10 weeks. However, studies have demonstrated that the continuation of weekly training following an initial strength training programme can help to maintain muscle strength and mass in older individuals [32]. Hägglund et al. [24] also observed a training effect after a 5-week intervention period and, based on other studies, reported a normalised swallowing rate after 5–8 weeks of neuromuscular training and at least an increase in swallowing rate after 13 weeks of training. Besides this ongoing training may support the maintenance of swallowing function. This underscores the need for a prolonged training period. This highlights the need for objective outcome measures, a control group and clearly defined variables in future studies.

The scheduling of morning training sessions was met with high satisfaction among residents. Likewise, SLTs observed heightened attentiveness among participants during this time. The existing literature, however, presents mixed findings regarding time-of-day effects. Some researchers have reported positive cognitive outcomes among older adults participating in afternoon [33], while others have found no significant differences in cognitive effects between morning and afternoon sessions of physical activity [34].

Furthermore, the questionnaire did not explicitly assess satisfaction with the mobilisation exercises or session closings involving group singing, as these were not formally designated as exercise components. Nevertheless, in the interviews, the SLTs commented on both activities, noting that song selection was consistently tailored to residents’ preferences. Indeed, recent studies have demonstrated the efficacy of personalised music therapy interventions for individuals with mild cognitive impairment [35]. In this regard, listening to music evokes positive responses such as attentive listening [35]. Taking into account that most of the residents have hearing impairments, increasing attentive listening could be a beneficial aspect.

The literature highlights a link between facility size and intervention success or functional outcomes among residents of LTC facilities. For example, Li et al. [36] found an association between high-volume facilities – defined as those with an average of more than 101 residents – and reduced functional decline, possibly attributable to the organisational efficiency and diversity of services provided by larger facilities. In the present study, a positive correlation emerged between facility size and resident satisfaction. One potential explanation for this could be the superior personnel resources at larger facilities, which may support the coordination and delivery.

SLTs identified a range of effects extending beyond the specific intervention content, noting residents’ heightened self-awareness of personal abilities and limitations, as well as social and emotional enrichment. Awareness of swallowing and speech difficulties, both in oneself and in others, is vital for enabling timely interventions and preventing more serious complications. Research has indicated that older adults frequently fail to recognise or report swallowing difficulties [24, 37] potentially due to the widespread belief that such issues are untreatable [24]. In this vein, SLTs indicated that the educational components of the intervention sometimes contributed to shifting residents’ perceptions of their own limitations.

SLTs also observed important social and emotional changes among residents, reporting that many residents appeared to develop increased self-confidence over the course of the intervention. Possibly, this development was supported by opportunities to be the centre of attention during both biographical conversations and participant-led closing activities, such as the recitation of poems or/and the singing of songs. Moreover, increased social interaction was also observed, even outside of the intervention setting. The literature supports the idea that group leisure activities can improve well-being in LTC facilities. Furthermore, the efficacy of reminiscence therapy in mitigating depressive symptoms – which affect approximately 50% of individuals requiring care – has been well documented [38]. According to SLTs, the final communicative sequence of each session often prompted discussions of emotional topics. As noted in the literature, such discussions can facilitate the expression of personal feelings and opinions, while providing group members opportunities to develop and demonstrate their communication skills [39].

In implementing the programme, SLTs were required to address various challenges and make adjustments at both individual and group levels, guided by their professional expertise. Similar situations are likely to arise in future implementations and must be managed with competence. Research has demonstrated that residents of LTC facilities respond particularly well to interventions that consider their health status, emotional well-being and cognitive abilities [40]. In this respect, further research is needed to determine the specific competencies (e.g., group management) required for SLTs to successfully and effectively deliver this intervention.

### Strengths and limitations

The triangulation of quantitative data from residents and qualitative data from SLT interviews enabled a multi-perspective view of the preventive OrkA intervention, providing unique insights into the challenges, strengths and effects of preventive group training. To date, only a limited number of interviews with therapists have been conducted regarding similar interventions. Thus, the present qualitative interviews contribute extensive new findings in an area that has otherwise received relatively little research attention. Moreover, the temporal proximity of the interviews – conducted immediately after the conclusion of each group intervention – enabled SLTs to provide detailed and reflective accounts (as substantiated by the high degree of data saturation achieved). This comprehensive feedback on aspects such as feasibility, challenges, limitations and benefits – combined with quantitative data on residents’ satisfaction – supports further development of the intervention manual.

The usability of the findings is limited, as they cannot be generalised to other curative approaches beyond the scope of the OrkA intervention. Moreover, the results derived from the OrkA project imposed constraints on both the number of SLTs available for interview and the geographical scope of the intervention (restricted to the Hanover region). As stated in the article, the SLTs surveyed were both trainers of the intervention and part of the research team. This may have led to selection bias. On the one hand the residents developed a personal professional relationship with the trainers after the 24 sessions, which may have influenced their responses to the satisfaction survey (social desirability). On the other hand, only those who completed the training programme responded to the satisfaction survey (nonresponse bias). There is no feedback from those who dropped out. The evaluation carried out by one of the trainers may also have led to confirmation bias, whereby the data was interpreted in a way that confirmed existing beliefs. Moreover, given the close professional relationships between the SLTs and WW and SM, the interview content may be subject to bias. Similarly, positive responses to the resident feedback questionnaires may have been influenced by favourable relationships with the SLTs or by social desirability bias. Notwithstanding these limitations, the data nonetheless inform and support the work of broader research practice, offering indicative trends relevant to the development of other group programmes in LTC facilities.

## Conclusion for practice

The aim of this paper was to establish and improve the preventive group intervention through assessed residents’ satisfaction and additionally perceived feasibility and usefulness from the complementary perspective of the administering SLTs.

Overall, resident and SLT results indicated that the intervention was perceived favourably by both parties. However, feedback suggested a need for adjustments to the swallowing and language exercises, the materials (e.g., worksheets) provided as well as the design and use of volitional devices for the swallowing exercises. In particular, alternative solutions should be developed to accommodate physical barriers such as dentures, cervical spine issues, visual impairments and arthrosis. Further research is also required in the domain of self-directed learning resources, to support the optimal design of worksheets, volitional devices and enhance residents’ motivation for independent learning.

The SLTs’ impressions suggest that OrkA training had beneficial side impacts on some residents. The reported observations were not only related to the target parameters of swallowing and speech. The SLTs reported noticing changes in learning motivation, mood and increased communication and social interactions. Although these observations are limited to a qualitative level, they provide valuable insights for further research, in which the abovementioned changes should be evaluated quantitatively in terms of their statistical effect due to such interventions.

## Supplementary Information

Supplementary Material 1.

## Data Availability

All anonymised data generated or analysed during this study are available from the corresponding author upon reasonable request.

## Abbreviations

LTC: Long-term care
cRCT: Cluster randomised controlled trial
MC: Main category
n: Sample size
OrkA: Orofacialpharyngeal and communicative activation in old age
CFIR: Consolidated framework for implementation research
p: Probability
r: Correlation coefficient
SC: Subcategory
SD: Standard deviation
SLT: Speech and language therapist
SPSS: Statistical Package for Social Sciences
VHW: Voluntary homework
